# Moderate heritability of hepatopancreatic parvovirus titre suggests a new option for selection against viral diseases in banana shrimp (*Fenneropenaeus merguiensis*) and other aquaculture species

**DOI:** 10.1186/s12711-016-0243-8

**Published:** 2016-09-07

**Authors:** Chontida Phuthaworn, Nguyen Hong Nguyen, Jane Quinn, Wayne Knibb

**Affiliations:** University of the Sunshine Coast, Locked Bag 4, Maroochydore DC, QLD 4558 Australia

## Abstract

**Background:**

In shrimp farming, major production losses are caused by viruses. Hepatopancreatic parvovirus (HPV) is one of the viral pathogens that infect banana shrimp (*Fenneropenaeus merguiensis*). HPV is thought to slow down growth and cause mortality in the juvenile stages of banana shrimp. Genetic improvement through selection of shrimp resistant to viral diseases is one approach to address this issue. This is the first detailed report on an aquaculture species that investigates whether viral titre varies among families and is heritable, and thus whether viral titre per se is a possible candidate trait for selection to produce resistant stock.

**Results:**

HPV titre was measured by quantitative polymerase chain reaction of DNA extracted from 1137 offspring (from 48 full-sib families). Estimated heritability of HPV titre, based on the linear animal mixed model, was moderate (h^2^ = 0.41). Genetic correlations of HPV with body traits (weight, length and width of body, head and tail) ranged from −0.13 to −0.38. HPV titre was negatively correlated with raw and cooked body colour (−0.33 and −0.43, respectively).

**Conclusions:**

This is the first study based on a large dataset that provides evidence that viral titre may have a genetic component in penaeid shrimp or even in any aquaculture species. The moderate heritability estimated for this trait suggests that resistance to HPV may be achieved by selecting for low HPV titre. With moderate and negative correlations, selection for resistance to HPV should gradually improve body traits and colour of banana shrimp.

## Background

Hepatopancreatic parvovirus (HPV) is a viral pathogen that infects penaeid shrimp and also frequently co-infects shrimp with other viruses [1–3]. HPV was reported to cause mortality in the early life stages of shrimp and to stunt growth at juvenile stages in grow-out ponds [4, 5]. In addition, HPV infection was suggested to slow down growth rates because of the observed negative correlation between HPV severity index and shrimp body length [2, 5]. The slowed down growth results in undersized shrimp at harvest time and thus, reduces either quantity or live weight of total production, and consequently contributes to profit loss.

Various biosecurity practices in hatchery and farming systems, e.g., pathogen screening in broodstock, water filtration and disinfection before stocking, have been advocated to control the spread and impact of shrimp viruses [6]. On the one hand, information on the origins and reservoirs of viruses is often lacking and prevents the use of appropriate tools and methods to detect and prevent infections. On the other hand, vaccination against bacteria and viruses has been used in commercial fish farming, especially in salmonids [7, 8]. However, such measures require an adaptive immune system and penaeid shrimp are thought to have only an innate immune system, precluding vaccination as an option to prevent viral infection in shrimp.

In addition to the biosecurity practices, genetic improvement is another option to control the impact of viral diseases by selecting for disease resistance [9]. The main goal of selection for resistance to viral diseases is to develop breeding lines which are less susceptible to viral infection, or at least that tolerate the effects of infection until harvest. Whether such genetic progress can be obtained depends in part on the magnitude of the additive genetic variation (i.e. heritability) of the trait [10, 11].

Heritabilities of resistance against viral diseases have been reported in several aquaculture species. In farmed fish, estimated heritabilities of resistance to viral infection are generally moderate to high and can range from 0.24 to 0.79 [9–12]. In marine shrimp, heritabilities of resistance to viruses have been reported mainly for pacific white shrimp (*Litopenaeus vannamei*), i.e. against taura syndrome virus (TSV) [13, 14] with moderate heritabilities (h^2^ = 0.22 to 0.30) and white spot syndrome virus (WSSV) [15, 16] with very low heritabilities (h^2^ = 0.01 to 0.07).

To date, heritabilities of viral resistance in aquaculture species have been estimated by using survival data after challenge testing. Survival data was considered as a binary variable (i.e. 0 = dead and 1 = alive) that was recorded at the end of the experiment, usually at the time when 50 % of the tested animals are dead or mortality rates flatten out [10, 11, 15]. A classic linear model (a cross-sectional model) is commonly used to handle the binary survival data for genetic estimations; however, this approach does not consider other information in the data such as time until death and other quantitative measures [17]. To better estimate genetic parameters of survival data from challenge testing, a number of more advanced models have been developed, including the threshold model [18], proportional hazard frailty model [19], linear repeatability model [20] and cure model [21]. Such models have been used to predict genetic parameters of survival data in various aquaculture species [9, 11, 16, 22]. For survival from infectious salmon anaemia (ISA) in field outbreaks, the predictive ability of the linear repeatability model was enhanced by 10 to 12 % compared to the classic cross-sectional linear model [23].

Although many comprehensive models have been developed to increase the accuracy of genetic estimates from survival data, setting an experimental infection to obtain survival information still requires a dedicated testing design that considers inoculation approaches, challenge dosage, stock density, testing capacity and operational costs [15]. All intensive attempts to optimize challenge conditions aimed primarily at obtaining records that can represent resistance mechanisms close to those under commercial conditions [16]. Resistance to virus has long been defined as survival from challenge testing, and is commonly applied in farmed fish. However, for shrimp there were concerns on how well survival to acute challenge testing reflects the innate genetic ability to resist to viral diseases because of the lack of a standard protocol for challenge conditions [15, 16]. Lack of standard and repeatable dosages during challenges may contribute to the observed low heritabilities.

An alternative to measuring the phenotypic impact on the host of the virus is to measure the viral titre itself, so that viral titre may be the selection criterion, provided it is heritable. Indeed, Van Koevering et al. [24] and Bosio et al. [25] reported that virus titres are heritable traits, i.e. for spindle streak mosaic virus in wheat and dengue-2 virus in mosquito, respectively. In the current study, we propose, for the first time, a new approach and selection criterion for an aquaculture species i.e. shrimp, which consists in simply measuring viral titre within and among shrimp families. This approach may have several advantages over classical challenge testing and may overcome many of the complications that arise from classical challenge testing. The first possible advantage is that measurements of viral titre can be quantitative, which should add power and resolution to the analyses and selection. Second, viral titre may reflect the innate ability to resist to viruses rather than an ability to survive. There are also a number of other appealing aspects of using viral titre as a measure for viral resistance which have been discussed in our previous study [26]. For example, it does not require a challenge facility to obtain viral titre data; the data can be obtained directly from commercial operations, which makes it highly relevant and applicable to commercial conditions. The high relevance of such viral titre data to commercial conditions can be important especially in shrimp farming systems for which the effectiveness of routine biosecurity practices is hampered by the limited available knowledge about the nature of the viruses (as mentioned above), and thus, often, viral infections cannot be effectively avoided. The lack of need for dedicated experimental challenge facilities reduces costs due to testing operations and facility maintenance. The opportunities to select for viral titre under full commercial conditions apply routinely to shrimp farming in Australia where the future broodstock are taken from commercial grow-out ponds; i.e. broodstock can be selected from the same pond as the animals tested for viral titre based on records of their full-sibs; however such opportunities may not apply so well to other industries that do not take their broodstock from commercial production systems.

One may postulate that using viral titre as a selection criterion irrespective of its heritability cannot yield improvement in survival, due to poor correlations between survival and viral titre and thus that classical challenge testing cannot be replaced by screening for virus titre. However, from a logical point of view, the idea that selection of resistant individuals will reduce or eliminate virus titre and thus, reduce or eliminate the negative impacts of a given virus on a given aquaculture species, is appealing.

Overall, in view of all these potentially desirable aspects of using viral titre as a selection criterion, and notwithstanding the efficacy of viral titre versus survival as a selection criterion, it is remarkable that, until this report and that of Knibb et al. [26], there have been no attempts to estimate the heritability of viral titre in an aquaculture species. Here, we address this knowledge gap by using a large dataset and viral copy number as a quantitative measurement to detect putative genetic resistance to HPV and to estimate the heritability of viral titre and its genetic correlations with body, carcass and flesh quality traits in banana shrimp.

## Methods

### Animal samples

Hepatopancreas samples of 1137 banana shrimp were collected at harvest time after 140 days of grow-out; shrimp had an average body weight of 16.5 g. These 1137 individuals were the offspring of 48 full-sib families with family size ranging from 9 to 30 offspring. They were cultured within a single grow-out pond in a commercial banana shrimp farm at Cardwell, Australia. Details on rearing conditions, breeding practices, genotyping and pedigree construction are in [27, 28]. The genotypes, based on DNA microsatellites, were used to assign animals to full-sib groups and, thus to establish the pedigree.

### Quantitative polymerase chain reaction

Essentially standard curves were produced using known DNA concentrations, and the DNA amounts in the new samples, which were estimated with reference to the standard curve. The genomic DNA of 1137 banana shrimp was extracted using a DNeasy Blood and Tissue Kit (QIAGEN) following the manufacturer’s instructions. DNA concentration was measured using a NanoDrop 2000 spectrophotometer (Thermo Fisher Scientific), and normalised to a concentration of 800 ng/μL. HPV titres were quantified based on an absolute quantitative PCR method using the SensiMix HRM kit (Bioline) and validated HPV primers, HPV140F (5′-CTACTCCAATGGAAACTTCTGAGC-3′), HPV140R (5′-GTGGCGTTGGAAGGCACTTC-3′) [29]. Quantitative polymerase chain reaction (qPCR) reactions were performed with a Rotor-Gene 6000 thermal cycler (Corbett Research). Details about standard curve, components of the qPCR reaction mix and cycling conditions are in [26]. Briefly, a standard curve was generated based on tenfold serially-diluted known concentrations of purified HPV PCR products. A final volume of 10 μL reaction mix contained 1 × SensiMix, 0.4 μL Evagreen dye, 3.5 mM MgCl_2_, 250 nM primers and 4 μL (i.e. 160 ng) normalised DNA. Cycling conditions were as follows: a first step at 95 °C for 10 min, 35 cycles at 95, 60 and 72 °C each for 25 s. The HPV titres were expressed as copy number per μg DNA based on the standard curve.

The hepatopancreas samples were separated into two main groups. The first group consisted of 897 samples for which HPV titres had not been quantified. The second group included 240 samples for which HPV titres had been determined in our previous study [26]. In this study, HPV titres of 135 samples in the second group were quantified for a second time, whereas the remaining 105 samples (that represented five qPCR batches from our previous work) were simply used in the statistical analyses. In the current study, 20 qPCR batches were assayed over a period of five months for HPV detection with an average of 52 samples per batch. In each batch, samples from at least 23 different families were randomly selected. In total, there were 25 qPCR batches represented in the subsequent analyses, 20 batches from the current study and five batches from the previous study [26].

In our previous study [26], each sample was analysed two to three times (replicates) and average HPV titres were used in the statistical analyses. In the current study, we re-analysed 135 samples from the earlier study to verify the consistency of the HPV results. Quantifications of the HPV titre of the 135 samples were conducted once (no replicates). Our statistical analysis showed that there was a very high, close to 1, correlation between the two studies. Statistical data indicated that measuring each sample once was sufficient to determine the HPV titre accurately. Thus, in this study, HPV titres of 897 samples that had not been previously quantified, were obtained from a single HPV titre quantification. As a further ongoing check for consistency of HPV titres, each new batch of tests included in duplicate either three random samples selected from the previous batch, or three known samples having a low [log(HPV) ~ 3], moderate [log(HPV) ~ 5], and high HPV titre [log(HPV) ~ 8], respectively.

### Statistical analyses

The shrimp HPV titres that were quantified by qPCR were transformed to the base 10 logarithm termed log(HPV). All preliminary statistics were analysed using the IBM SPSS Statistics 22 software [30]. The log(HPV) values for shrimp samples that were higher than 3 standard deviations of the mean (${\bar{\text{x}}} \, \pm \, 3\sigma$) were considered as outliers, and excluded from the statistical analyses. The observed ${\bar{\text{x}}} \, \pm \, 3\sigma$ of log(HPV) was 4.74 ± 3.81, ranging from the minimum value of 0.93 to the maximum of 8.55. Log(HPV) values for shrimp samples were all within the permitted range, which indicates that there were no outliers in the data, and thus no HPV titres for the tested shrimp were excluded from statistical analyses.

We used histograms to graphically represent the distribution of the HPV titre data and assess its normality. The empirical frequencies of log(HPV) followed the theoretical curve drawn from a normal distribution, which indicated that the HPV titre data was approximately normally-distributed. ANOVA analysis was conducted to examine statistical differences of log(HPV) means between the 48 families studied. The general linear model was used to investigate the relationships between fixed factors and log(HPV). The fixed factors included sampling locations around the pond, sampling time, the person who sampled the hepatopancreas, sex, DNA quality, qPCR batch and qPCR run date. Among these factors, only the effect of qPCR batch was statistically significant (*P* < 0.05) and was included in the model to estimate heritability of log(HPV). The proportion of log(HPV) variation explained by the qPCR batch in the statistical model was only 3.8 %.

### Mixed model analyses

#### Heritability (h^2^)

The heritability of log(HPV) was estimated using a linear animal mixed model in ASReml 4.1 [31]. The model was defined as:

1 $${\text{Y}}_{\text{mn}} = \upmu + {\text{Q}}_{\text{m}} + {\text{a}}_{\text{n}} + {\text{e}}_{\text{mn}} ,$$

where Y_mn_ = log(HPV), μ = estimated population mean, Q_m_ = qPCR batch; m = 1–25, a_n_ = additive genetic effect of individual shrimp in the pedigree; n = 1 to 1137, e_mn_ = residual.

#### Phenotypic (r_p_) and genotypic (r_g_) correlations

The phenotypic and genotypic correlations of log(HPV) with commercial traits such as body weight, body length, head length, body width, abdominal tail weight, meat yield, raw and cooked colour, and ‘flesh streaks’, were estimated using a series of two-trait animal mixed models in ASReml 4.1 [31]. ASReml enabled the specification of different effects for different traits in the statistical models. For body and flesh quality traits, the model included the fixed effects of sampling batch, sampling time, operator and sex [28]. The effect fitted for log(HPV) was only qPCR batch as described in Eq. 1. The general form of the two-trait model was defined as:

2 $${\text{Y}}_{\text{ijklmn}} = \upmu + {\text{S}}_{\text{i}} + {\text{T}}_{\text{j}} + {\text{O}}_{\text{k}} + {\text{S}}_{\text{l}} + {\text{Q}}_{\text{m}} + {\text{a}}_{\text{n}} + {\text{e}}_{\text{ijklmn}} ,$$

where Y_ijklmn_ = traits under study, μ = estimated population mean, S_i_ = sampling batch within a single grow-out pond; i = 1–8, T_j_ = sampling time; j = AM and PM, O_k_ = operator; k = 1 and 2, S_1_ = Sex; l = M and F, Q_m_ = qPCR batch; m = 1–25, a_n_ = additive genetic effect of individual shrimp in the pedigree; n = 1 to 1137, e_ijklmn_ = residual.

### Testing different analysis models for estimating heritability (h^2^)

In this study, heritability of log(HPV) was estimated by using the model that included the significant effect i.e. qPCR batch (Eq. 1). In addition, we fitted a different model that included other fixed effects as described in Eq. 2 to estimate the heritability of log(HPV). The statistical difference in heritability estimates between the two models was examined by using the z-score [32]:

$${\text{z}} = \frac{{{\text{x}}_{\text{i}} - {\text{x}}_{\text{j}} }}{{\left( {\upsigma_{\text{i}}^{2} + \upsigma_{\text{j}}^{2} } \right)^{0.5} }},$$

where x_i_ and x_j_ are the estimated heritabilities based on Eqs. 1 and 2, and σ_i_ and σ_j_ are their respective standard errors. The observed z-score was tested against a large-sample normal distribution (α = 0.05).

## Results

**Fig. 1:**
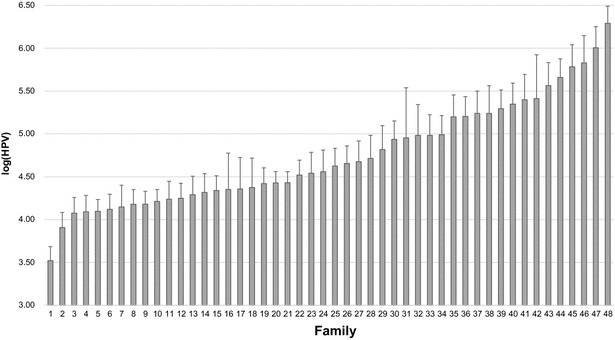
Average log(HPV) showing significant differences (*P* < 0.001) across the 48 full-sib shrimp families

**Fig. 2:**
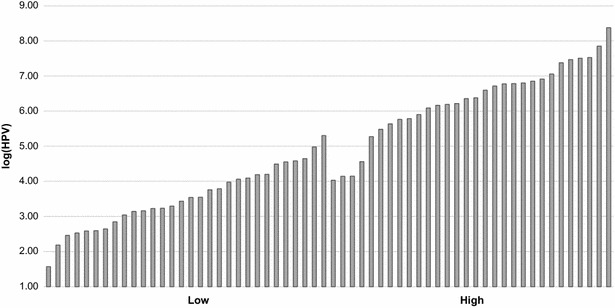
Variation in log(HPV) among individual shrimp within a family. *Low*: family with the lowest average HPV titre (i.e. the highest HPV resistance); *high*: family with the highest average HPV titre

The log(HPV) values averaged for each of the 48 families ranged from 3.52 ± 0.16 to 6.29 ± 0.20, and showed significant differences across families (ANOVA F_47,1089_ = 7.334, *P* < 0.001; Fig. 1). These significant differences in log(HPV) across families were also confirmed by the log-likelihood ratio test (*P* < 0.001). The log(HPV) of the family with the highest HPV titre was more than 2.5 fold higher than that of the family with the lowest HPV titre. There were also large differences, up to sixfold, in log(HPV) among individual shrimp within families. An example of log(HPV) variation within the family with the lowest (i.e. highest HPV resistance) and that with the highest HPV titre is in Fig. 2.

### Reproducibility and correlation of qPCR results

**Fig. 3:**
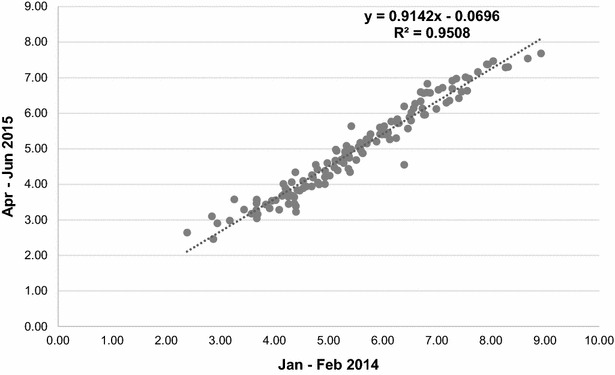
Scatter plot of the linear relationship of log(HPV) between the previous [26] (N = 135) and present study (N = 135).* X*-axis (Jan–Feb 2014) represent qPCR results from the previous study and *Y*-axis (Apr–Jun 2015) qPCR results for the same samples obtained in the current study

A subset of 135 of the 240 samples that were previously assayed was re-analysed in this study. The correlation of HPV titres between the two studies was highly significant (*P* < 0.001) with a correlation coefficient (*r*) close to 1, *r* = 0.98, (Fig. 3). This consistency between both studies indicated a high level of accuracy in the quantification of HPV titres. In spite of the high reproducibility of HPV titres between both studies, we still expected some differences due to systematic factors such as qPCR being done by different persons, on different dates or times, and qPCR-reaction-mix preparation. In this study, we considered the combination of these factors as the effect of qPCR batch.

### Heritability (h^2^) and correlations (*r*) between traits

The heritability of log(HPV) was moderate, i.e. 0.41 ± 0.08 (Table 1), which indicated that this trait was heritable and could be potentially improved by selective breeding. Phenotypic (*r*_p_) and genotypic (*r*_g_) correlations of log(HPV) with body and carcass traits are in Table 2. Phenotypic correlations between log(HPV) and the other traits studied were low (−0.01 to −0.14), whereas genetic correlations were generally moderate and ranged from −0.13 to 0.5. Phenotypic and genetic correlations of log(HPV) with body and carcass traits were negative except those with meat yield which were positive. The genetic correlation between log(HPV) and yellow hepatopancreas was moderate and positive. All estimated genetic correlations had greater magnitudes than phenotypic correlations.

**Table 1:** Number of samples (N), mean, standard deviation (SD) and heritability (h^2^) for HPV titre

Trait	Unit	N	Mean	SD	h^2^ ± SE
HPV titre	Copy number per μg DNA	1137	3.10 × 10^6^	1.57 × 10^7^	
log(HPV)		1137	4.74	1.27	0.41 ± 0.08

**Table 2:** Phenotypic (*r*_P_) and genetic (*r*_g_) correlations of log(HPV) with commercial traits

Traits	*r*_P_	*r*_g_
Body weight (g)	−0.14 (0.04)	−0.33 (0.15)
Body length (cm)	−0.17 (0.04)	−0.38 (0.14)
Head length (cm)	−0.07 (0.03)	−0.13 (0.18)
Body width (mm)	−0.15 (0.04)	−0.35 (0.15)
Abdominal tail weight (g)	−0.13 (0.04)	−0.31 (0.15)
Meat yield (%)	0.07 (0.03)	0.50 (0.20)
Colour of raw shrimp	−0.10 (0.03)	−0.33 (0.17)
Colour of cooked shrimp	−0.06 (0.03)	−0.43 (0.18)
‘Flesh steaks’	−0.03 (0.03)	0.37 (0.31)
Yellow hepatopancreas	−0.01 (0.03)	−0.40 (0.42)

Standard errors in parentheses

### Effect of using different models on heritability (h^2^)

The additional analysis that included other systematic effects in the model (Eq. 2) yielded heritability estimates for log(HPV) that were very similar to those based on the model that included only the effect of qPCR batch (Eq. 1) (0.403 ± 0.080 versus 0.410 ± 0.080, z = 0.097, *P* > 0.05).

## Discussion

### Reproducibility and feasibility

Overall, we found that qPCR for HPV detection in banana shrimp was highly reproducible when the same sample was tested even months apart. Such high reproducibility of qPCR has been reported elsewhere [33, 34] and this is of practical importance since the estimation of genetic parameters requires a large number of samples, and many months of analyses.

The current work was completed over several months and faster turnaround is probably needed for applied selection programs. However, recent technologies and qPCR machines that are capable of analysing 96 samples, even up to hundreds of samples at a time, have become available, at relatively low costs; our calculations are that the same work can now be completed in weeks instead of months. DNA assays such as immunofluorescence titration and virus particle counts are fast and inexpensive methods to measure viral titres, although perhaps at the cost of accuracy [24, 25]. Moreover, should interest shift from challenge testing to viral titre as selection criterion, it is also anticipated that the identification of single nucleotide polymorphisms (SNPs) specific to full-sib families with low or high HPV titres from differential gene expression analyses [35] would be a useful alternative since SNPs represent an even lower-cost selection criterion; however, to date, quantitative trait loci (QTL) for HPV resistance have not been identified.

### Substantial variation among families and moderate heritability

This is the first report, apart from our preliminary study [26], which provides an estimated heritability for viral titre in an aquaculture species. In contrast to the previously reported low estimated heritabilities for survival to virus challenge in shrimp, our estimated heritability for viral titre was moderate. It is a little premature to suggest that a moderate heritability would be the general expectation from future similar studies in different species and with different viruses. However, there are several discussable aspects of our approach, which suggest that it may yield higher heritabilities than those based on survival data from challenge testing in shrimp. For example, on the one hand, survival data may be influenced by substantial uncontrolled environmental variation to the challenge testing conditions (e.g. stock density and infection dosages due to variable feeding) [15]. We found a high level of reproducibility in the quantification of viral titre for given animals and the between-batch effects contributed to less than 5 % of the variation in viral titre. On the other hand, there is the potential for much uncontrolled environmental variation in viral titre among shrimp in the ponds, and thus, natural outbreaks may have more variable environmental effects than controlled challenge tests, but the *prima facie* evidence of a moderate heritability indicates that this uncontrolled variation was not sufficient to obscure genetic effects. Another advantage of using viral titre from qPCR analysis to assess resistance to virus is that shrimp samples can be directly collected at harvest times under farm condition, and transferred to the laboratory for viral quantification. This procedure of testing the animals from commercial conditions does not require challenge facilities, thus investments related to challenge testing (e.g. challenge facilities, testing and maintenance) are reduced or even completely avoided. This approach also guaranties that the assessed parameters are relevant to commercial operations, whereas there is always concern about whether dedicated challenge testing is fully relevant to commercial production. The moderate heritability of viral titre suggests that selective breeding for low viral titre is possible, and may result in shrimp that are resistant to the virus.

Not only was there substantial HPV titre variation among families, there was also substantial variation among individuals within each family, which suggests that selection should consider both within- and between-family variation.

### Genetic associations between HPV titre and commercial traits

The genetic correlation between HPV titre and body weight was negative and the magnitude of the correlation was moderate (−0.33), which suggest that selection to increase HPV resistance (i.e. by reducing HPV titre) would improve growth performances (e.g. increase body weight) in banana shrimp and vice versa. Our results contrast with those of a previous study on pacific white shrimp for which highly negative genetic correlations between body weight and WSSV resistance were found for two breeding lines (−0.55 and −0.64) [15]. In marine fish breeding, genetic correlations between body weight and resistance to pathogens vary according to the infective agent. Low genetic correlations (*r*_g_ ~ 0) were reported between body weight and resistance to viruses and bacteria, e.g., viral nervous necrosis (VNN), vibriosis and furunculosis in various cultured marine fishes [36–38]. However, a moderately negative genetic correlation (−0.32) was reported in Atlantic salmon between body weight and resistance to parasitic disease, *C. rogercresseyi* [39], while adverse genetic correlations of body weight with resistance to viral haemorrhagic septicaemia (VHS) were low to moderate (−0.14 to −0.23) in rainbow trout (*Oncorhynchus mykiss*) [40]. To some degree, the genetic correlations may depend on how the data are collected: growth measured on infected animals may be a different trait to growth measured on uninfected animals, and the former may rather be a measure of disease resistance than growth *per se*.

Improving HPV resistance in shrimp is also expected to increase some carcass characteristics (i.e. abdominal tail weight) as indicated by the negative (favourable) genetic correlations between the two traits. Besides growth performances and carcass characteristics, raw and cooked colours of banana shrimp are expected to be improved (more pronounced dark or red colour) by selecting for HPV resistance. Another finding of our study was the negative genetic correlation between HPV titre and condition of the hepatopancreas (yellow hepatopancreas). This could be a desirable trait from a commercial perspective because selection for reduction in HPV titre (i.e. increase in HPV resistance) will likely decrease the incidence of shrimp with yellow (presumably undesirable) hepatopancreas. Unfortunately, to our knowledge, genetic correlations between viral resistance and condition of the hepatopancreas have never been reported in any other aquaculture species for comparison with our findings.

To date, the nature of the defence mechanisms against viruses in shrimp is still unclear. Various studies [1–3, 41] have reported that HPV was co-expressed with other viral diseases, frequently with monodon-type baculovirus (MBV), WSSV and infectious hypodermal and hematopoietic necrosis virus (IHHNV), but there is no report on genetic correlations between HPV and other viral diseases. This is an important knowledge gap, since selection against one virus may confer resistance to others.

The favourable heritability of HPV titre and genetic correlations that we obtained in this study indicate promising opportunities to improve HPV resistance in banana shrimp without compromising other important traits.

### Association of HPV titre with survival

In this study, we do not report any hard data that specifically relate HPV titre to survival. What data that are available are only circumstantial. For example, as reported above, we observed negative genetic and phenotypic correlations of HPV titre with measures of growth; this suggests that HPV titre has an impact on production traits but may not particularly relate to survival. There are data on counts of offspring per family but they likely reflect problems of differential fertility and other genetic or stochastic events in the hatchery and prior. Indeed these counts were not significantly correlated with family means of log(HPV).

One unresolved matter is the degree to which HPV titre and survival are correlated, or not, and whether such a correlation is evident only at a certain threshold titre, i.e. perhaps below this threshold there is no correlation; shrimp may have HPV but are not “sick” or “diseased” or do not show any specific clinical signs. Indeed, various studies [1, 4, 5] have reported that HPV-infected shrimp do not show gross signs of disease and act normally; only impacts on stunt growth in juveniles and mortality in severely-infected post-larvae were observed. In addition, Flegel et al. [2] reported that most black tiger shrimp from commercial grow-out ponds were positive for HPV, while no ponds showed an unusual level of mortality. Therefore, it is likely that moderately HPV-infected shrimp cannot be readily identified just by external appearances; only severely-infected shrimp may present symptoms. Manivannan et al. [1] reported that the symptoms of severe infections in juvenile shrimp were a whitish hepatopancreas, poor growth rate, anorexia, gill fouling and susceptibility to surface as a result of reduced preening activity. Based on literature data, it is hypothesized that health consequences may vary according to HPV titres, which is consistent with some industry operating views and procedures. The industry considers that ponds with high HPV titres risk catastrophic collapse when they reach the threshold at which HPV impacts health, but ponds are harvested well before the onset of collapse, as would be the case in our experiment. Thus, one can argue about whether HPV titres had reached a sufficient level for major impacts to occur on either individuals or the whole pond. Another complication in associating HPV titre with survival *per se*, is that HPV is often detected with other viruses [3, 41]; mortality may not reflect specifically that due to HPV since the disease may be essentially due to other viruses.

To what degree many of these uncertainties and unknowns can be best resolved by returning to more classical challenge testing is debatable. Perhaps we could test predefined HPV titres against ensuing survival; this approach presumably would identify any effects of HPV titre and relate the effects specifically to HPV rather than to other viral infections. However, we would then be returning to the swath of issues and problems with challenge testing referred to in the discussion such as costs and potential inadequacy with industry practices, etc.

In conclusion of this section, it is possible that selection for viral titre at the levels recorded here may not achieve desirable outcomes for situations of extreme viral titres and catastrophic collapse, i.e. correlation between HPV titres reported here and survival to HPV is poor as the pond evolves towards collapse. Notwithstanding these uncertainties, what we can say *ipso facto*, is that if we can eliminate the virus by selection, as suggested by this study, then concordantly, we will eliminate the negative effects of the virus.

### Mode of transmission (vertical or horizontal)

#### If transmission is horizontal

There are two major types of transmission, horizontal and vertical, although the two are not mutually exclusive. Horizontal transmission refers to all possible situations in rearing environments that diseases can be transmitted from infected animals to healthy ones in the same facility. There is circumstantial evidence for horizontal transmission of HPV: Catap et al. [42] detected infections in healthy black tiger shrimp (*Penaeus monodon*) after feeding animals with infected black tiger shrimp postlarvae, while Sivakumar et al. [43] reported that experimentally-infected live artemia given to healthy postlarvae of black tiger shrimp apparently caused infections. Other viruses (e.g. yellow-head virus (YHV) and WSSV) were also reported to be able to transmit and infect cultured shrimp horizontally [44, 45]. All these studies suggest that viruses can be transmitted either through infected live feed or by moribund shrimp being eaten due to the cannibalistic behaviour of shrimp.

In our study, shrimp were cultured communally in the same grow-out pond, thus presumably, they had equal chances to be exposed to diseases excepting prior infections due to vertical transmission. Variation in this type of (horizontal) transmission in the pond is more like an environmental factor, and by itself should not contribute to the additive genetic variation of HPV titre and the repeatable differences among families.

#### If transmission is vertical

There is a possibility that vertical transmission, from parents to offspring, may have contributed to the differences of HPV titres among families. Several previous studies investigated the potential for vertical transmission, from infected parents to offspring, of different farmed shrimp viral diseases, e.g., MBV and IHHNV. All these studies confirmed the existence of a parental contribution to viral infection in the offspring, however the proportion of virus transmitted to the offspring depended on the severity of the infection in the parents [46–48].

From a scientific perspective, vertical transmission if not swamped by horizontal transmission, could lead to inflated or completely artefactual genetic parameter estimates. Unfortunately, estimation of the maternal effect was not possible with our data structure and we cannot discount that the maternal effect from vertical transmission may have merged with, and inflated, the additive genetic estimates. In other words, there is considerable risk that the heritability is biased but that our design cannot avoid this pitfall. The only publications so far for farmed fish that are communally reared at early stages show that maternal effects are negligible, at least for traits such as growth and condition index [49, 50]. Such results contrast with those from terrestrial livestock for which the offspring after birth require maternal care and, thus, maternal effects are common [51–53]. Nevertheless, until further work is done, we cannot say if the pattern of a negligible maternal effect for farmed fish that are raised communally at early stages will also apply for viral infection in shrimp. This matter can be addressed in future studies that consider the use of maternal half-sib families, and by assessing the viral titres in the female broodstock.

While we have no direct evidence for vertical transmission of HPV in shrimp, if it occurs and contributes to the differences in HPV titres among families, then on-farm selection for families with low HPV titres will still result in lower HPV titres in the next generation through a reduction in the viral titre of the parents.

## Conclusions

The additive genetic variation of HPV titre suggests that resistance to HPV in banana shrimp can be improved by selective breeding. Selection for HPV titre may also improve other traits of commercial importance such as body weight, meat yield, condition of the hepatopancreas, and shrimp colour. This is the first major report on viral resistance as a quantitative trait and the genetic parameters of HPV titre, for an aquaculture species.
